# Perspectives in the Cell-Based Therapies of Various Aspects of the Spinal Cord Injury-Associated Pathologies: Lessons from the Animal Models

**DOI:** 10.3390/cells10112995

**Published:** 2021-11-03

**Authors:** Małgorzata Zawadzka, Anna Kwaśniewska, Krzysztof Miazga, Urszula Sławińska

**Affiliations:** Nencki Institute of Experimental Biology, Polish Academy of Sciences, 02-093 Warsaw, Poland; a.kwasniewska@nencki.edu.pl (A.K.); k.miazga@nencki.edu.pl (K.M.); u.slawinska@nencki.edu.pl (U.S.)

**Keywords:** spinal cord injury, cell transplantation, cell-based therapy, regeneration

## Abstract

Traumatic injury of the spinal cord (SCI) is a devastating neurological condition often leading to severe dysfunctions, therefore an improvement in clinical treatment for SCI patients is urgently needed. The potential benefits of transplantation of various cell types into the injured spinal cord have been intensively investigated in preclinical SCI models and clinical trials. Despite the many challenges that are still ahead, cell transplantation alone or in combination with other factors, such as artificial matrices, seems to be the most promising perspective. Here, we reviewed recent advances in cell-based experimental strategies supporting or restoring the function of the injured spinal cord with a particular focus on the regenerative mechanisms that could define their clinical translation.

## 1. Introduction

Spinal cord injury is a devastating neurological condition, leading to many severe deficits such as paralysis, sensory abolition, and respiratory, circulatory, and digestive system dysfunctions that usually result in permanent disability. The estimated global SCI incidence reaches 40 to 80 cases per million people and gradually increases with the rate of car or extreme sports accidents and in total. Costs of spinal cord injury are higher than those of comparable conditions such as dementia, multiple sclerosis, and cerebral palsy (WHO, https://www.who.int/news-room/fact-sheets/detail/spinal-cord-injury, accessed on 1 September 2021). Only in the United States, the annual incidence of SCI is estimated at approximately 54 cases per million people (17,730 new SCI cases each year) with an average annual cost of healthcare for SCI patients of 76,327 USD per year (National Spinal Cord Injury Statistical Center, Facts and Figures at a Glance. Birmingham, AL: University of Alabama at Birmingham, 2019, https://www.nscisc.uab.edu/, accessed on 1 September 2021). These data clearly indicate that SCI is not only a serious health condition that significantly reduces the quality of life, for both patients and their families, but also an important socio-economic problem, therefore an improvement in clinical treatment for SCI patients is urgently needed.

The cascade of pathological events within the injured spinal cord starts with tissue crushing or tearing caused by mechanical damage that leads to the disruption of the nerve fibers and blood vessels that run along the spinal cord, and damage of nerve and glial cells, tissue edema, and ischemia [1,2]. Such degradation of the neural tissue provokes an uncontrolled release of glutamate into the extracellular space, overactivation of NMDA receptors, and prolonged depolarization of neurons, resulting in their death [3,4]. Glutamate, acting via AMPA receptors, kills oligodendrocytes as well, which results in the demyelination of axons and, as a consequence, the death of unprotected neurons [5]. Primary injuries to the cord tissue lead to secondary damage, associated with tissue ischemia, hypoxia, and massively developing inflammatory reaction [6]. These further contribute to neural cell death via either necrosis or apoptosis, which increases the damage caused by primary insult. Soon after the spinal cord injury, a scar is formed around the trauma site. At the cellular level, the scar is very heterogenic and consists of many cell types: microglia- and monocyte-derived macrophages, reactive astrocytes, ependymal and endothelial cells, fibroblasts, connective tissue as well as a plethora of molecules produced by these cells such as collagens, laminins, fibronectin, tenascin-C, and periostin [7]. The scar that forms at the spinal cord injury site generally has “glial” and “fibrotic” components. The glial component of the scar refers to the reactive astrocytes that surround the central core and prevent peripheral immune cells from invading the CNS parenchyma. Fibrotic scars, on the other hand, are formed by fibroblasts, mostly of the perivascular origin, robustly distributed in the injury site, and an excess deposition of extracellular matrix molecules [8].

The scar rebuilds the blood–spinal cord barrier, protects healthy intact tissue, and limits further secondary tissue damage [9]. On the other hand, it creates a barrier for regenerating and sprouting axons [10]. In addition to the mechanical obstacle created by proliferating fibroblasts and astrocytes, the scar also builds a molecular barrier [11,12]. Many factors abundantly secreted by scar cells, such as chondroitin sulfate proteoglycans, the Nogo-A protein, or the myelin-associated glycoprotein (MAG), can bind to numerous receptors on the nerve cells to activate the RhoA/ROCK signaling cascade pathway that leads to a breakdown of the axon growth cone cytoskeleton [13].

The prevention and, if possible, reversal of these unfavorable secondary reactions is the ultimate goal of clinical strategies being developed for SCI treatment. Although many of the mechanisms responsible for the secondary injury cascade are already known and therefore could theoretically be targeted by pharmacologic treatment, these have not been clinically successful to date. Pharmacological approaches that have already been extensively tested in animal models have focused on the inhibition of neuronal death induced by glutamate excitotoxicity. This might be achieved by preventing the overactivation of glutamate receptors with their selective antagonist i.e., NBQX (a highly selective competitive antagonist of AMPA and KA ionotropic receptors) [14,15] or by preventing presynaptic glutamate release and blocking postsynaptic NMDA glutamate receptors with riluzole (an inhibitor for the kainite and NMDA receptors) [16]. Moreover, the reduction of glial scar formation via the administration of the iron chelator 2,2′-bipyridine, an inhibitor of prolyl 4-hydroxylase and a key enzyme of collagen biosynthesis [17], activation of the spinal cord neuronal networks that survived below the injury [18,19,20], and an increase in axon regeneration via inhibition of the RhoA/ROCK pathway [21] have been also postulated. On the other hand, modulation of the inflammatory response via a tetracycline antibiotic minocycline has been also proposed as a promising pharmacological approach. It has been shown that precise control of inflammation may protect neurons in the injured CNS [22].

However, the complexity and irreversible nature of the mechanisms involved in nerve tissue damage make it difficult to design an effective SCI pharmacotherapy. Therefore, exercise-based movement training coupled, or not, with spinal cord epidural electrostimulation remains the most efficient therapeutic procedure routinely used in SCI clinics. Experimental data from animal models show that its beneficial effect is mainly mediated by increasing the production of neurotrophins BDNF [23] and BDNF/NT-4 [24] within the spinal cord that, in turn, support neuronal survival [25] and plasticity [26,27]. On the other hand, locomotor training also exerts a positive effect on the growth of muscle mass and the overall condition of the body [28]. The beneficial effect of training, particularly in combination with electrostimulation, has been extensively experimentally tested and proven to enhance motor abilities, mainly by increasing activity and facilitating the plasticity of spinal cord networks. Spinal cord epidural electrostimulation was also proposed to cure the pain symptoms associated with SCI [29,30].

Recently, the development of cell-based regenerative approaches in medicine has revived the debate within the field and opened novel perspectives in SCI treatment. A wide range of cell types has been examined in animal models of SCI, including fully differentiated cells such as Schwann cells (SCs) [31], olfactory ensheathing cells (OECs) [32,33,34] as well as different types of stem cells, such as neural stem cells (NSCs) [35,36] and mesenchymal stem cells (MSCs) [37]. Cells can be grafted directly onto the injured spinal cord, often in scaffolds facilitating their survival [38], or applied intravenously [39]. The latter, however, could lead to cerebral embolism [40]. Therefore, more precise methods of cell delivery are currently being tested. The most sophisticated is, to date, an application of biocompatible and magnetically activable nanobots that have been designed to deliver cells precisely to the injury site [41].

Since whole-cell administration may likely be associated with certain clinical risks such as low cell survival rate, graft rejection, limited or improper differentiation, or tumor formation, novel approaches employing cell-derived structures or substances have recently been proposed. Among them, a very promising strategy employs small extracellular vesicles (sEVs), naturally released by cells to communicate with each other. sEVs can deliver many potentially neuroprotective molecules (such as miRNAs and proteins) directly to host cells of the injured spinal cord, affecting their activity and fate [42].

In this review, we discuss the recent achievements in exogenous cell-based restorative approaches that have been experimentally proven to be beneficial in rodent models of SCI with a special focus on their mechanisms of action in preventing diverse unfavorable changes caused by spinal cord trauma. The mechanism of action of exogenous cells in SCI treatment is mainly based on the modulation of the injured tissue microenvironment and/or replacing lost cells. Therefore, the greatest experimental efforts are mainly directed towards the inhibition of the inflammatory reaction that occurs after SCI, inhibition of neuron death, supporting axonal regeneration as well as rebuilding and/or activating surviving spinal cord neuronal networks.

## 2. Inhibition of Inflammatory Reaction

Inflammation is a natural immune response to tissue injury leading to control, clearance, repair, and eventually restoration of homeostasis. Upon spinal cord injury, neurons and other damaged cells release a plethora of inflammatory mediators activating various cell types including microglia, astrocytes, and endothelial cells. This further contributes to neuronal damage by initiating an inflammatory cascade in both the spinal cord and the immune system (for review: [43,44]). The earliest immune reaction to SCI is predominantly pro-inflammatory cytokine production by the recruited and proliferating microglia. Microglia are the innate immune cells of the CNS and, as such, they are sensitive sensors of any changes in homeostasis [45]. Once activated, they upregulate major histocompatibility complex class molecules and produce pro-inflammatory and cytotoxic cytokines, including tumor necrosis factor-α (TNF-α), the ligand for Fas receptor (FasL), and interleukin 1β (IL-1β) [46,47]. This leads to further immune system cell activation and a systemic defense reaction [48,49,50]. Cytokines produced by microglia also activate astrocytes, which in turn become important contributors to further inflammatory and immune responses. Reactive astrocytes proliferate, undergo hypertrophy, and produce neurotrophic factors, but also pro-inflammatory (IL-1, IL-6) and cytotoxic cytokines or reactive oxygen species and nitric oxide. Extensive astrocyte proliferation and hypertrophy lead to scar formation that may delay or inhibit regenerative responses [51]. However, genetic ablation of scar-forming astrocytes in adult transgenic mice results in neuronal degeneration [52] suggesting a beneficial role of reactive astrocytes in the injured nervous system. Inflammation often contributes to secondary damages after SCI. Excessive and prolonged production of pro-inflammatory cytokines leads to both neuronal and glial toxicity. Moreover, activated glial cells release lysosomal enzymes such as proteases as well as reactive oxygen species, which further impairs the injured tissue [49,50]. This harmful inflammatory response results in diminished axon regeneration, demyelination, cell death, and eventually a decrease in functional recovery.

Although inflammation and gliosis are hallmarks of the CNS pathology and targets of certain well-known anti-inflammatory drugs that could be potentially used in clinical SCI treatment, the appreciation of the beneficial side of neuroinflammation has initiated an extensive debate on the actual role of these processes in neurological disorders. Evidence that comes from the genetic or pharmacological inactivation of microglia/macrophages sheds light on the critical role of the timing and extent of microglial activation, distinct roles of different populations, and the beneficial role of inflammation in the regenerative phase of the post-injury response. Recent reports indicate that the response of microglial cells to the changes in CNS homeostasis is not uniform, and rather microglia and macrophages adopt a spectrum of phenotypes and functions, from pro-inflammatory to anti-inflammatory (for review: [53,54]). Soon after the injury, cells of myeloid origin, namely resident immune cells—microglia and blood-borne monocytes, recruited to the wound area—are converted into macrophages. Early-stage macrophages are usually primed to produce pro-inflammatory factors (classified as classical M1 type, for simplification) and during the resolution of inflammation, they are gradually replaced by cells polarized into the anti-inflammatory M2 type. The main role of M1 macrophages is removing tissue debris and microbes from the injury site, while M2 cells aim to heal the wound and remodel the surviving tissue. This process is critically disturbed during SCI, which is reflected, among others, by an elevated number and prolonged activity of pro-inflammatory cells [6]. Therefore, the concept of controlling microglia/macrophage activation and fine-tuning the microglial function in order to modulate them into the desired phenotype is currently considered a promising strategy for enhancing recovery in SCI treatment [55]. Specific attenuation of the detrimental effects of the prolonged inflammation and modulation of macrophage activation into a pro-regenerative state is a common mechanism of action of many types of stem cells transplanted into the spinal cord after injury. In the rat model of spinal cord compression, grafting mesenchymal stem cells into the area of injury caused an increase in the number of M2 macrophages and a reduction of M1 cells, leading to functional recovery of grafted animals manifested by higher Basso–Beattie–Bresnahan (BBB) hindlimb locomotor rating scores [56]. The enhanced polarization of macrophages into the M2 phenotype and the eventual reduction of inflammation in spinal mice can also be achieved by applying small extracellular vesicles (sEV) derived from MSCs [57]. On the other hand, intraspinal grafting of neural precursor cells in mice after a contusion spinal cord injury performed at the thoracic level [58], or in rats after a compression injury at the cervical level [59], caused downregulation of M1 macrophages with no significant impact on M2 macrophage activity. In grafted mice, functional recovery manifested by higher Basso Mouse Scale (BMS) scores were observed in subacute transplantation (7 days after injury) [58] but not in the case of transplantation at 21 days after contusion [60]. In rats, only minimal functional recovery manifested by a significantly larger footprint area in CatWalk XT analysis was observed, while BBB scores and the number of step errors in the grid walk test did not change significantly after cell transplantation [59].

It has been reported that the modulation of macrophage polarization could be achieved not only by grafting whole cells but also by the administration of a cell-conditioned medium. Both endothelial progenitor- and neural stem cell-conditioned media successfully mediated an anti-inflammatory effect and reduced the number of inflammatory macrophages in rodents with injured spinal cords [61,62]. NSC-derived anti-inflammatory factors positively regulating microglia activation and neuroinflammation may also be delivered to the injury site via isolated cell-derived small vesicles [42]. The transplantation of different types of exogenous cells into the injured spinal cord not only alters the inflammatory profile of macrophages but also regulates the response of other types of immune system cells. Neural precursor/stem cells grafting has been shown to increase T-lymphocytes production, with a reduced amount of B-lymphocytes and neutrophils in a mouse model of SCI [58,60].

At the molecular level, the immune reaction after SCI is a cascade of events driven by changes in the spinal cord environment, dependent on diverse groups of molecules, mainly chemokines and cytokines. They are involved in complex signaling pathways either activating or disabling immune cell function. Cytokine concentration can also be modified indirectly by regulating signaling pathways effectors, for example, nuclear factors. Transplantation of the human spinal cord immortalized neural stem cells into rats subjected to SCI resulted in the inhibition of NF-κB production and, as a result, modulation of the pro-inflammatory response [63]. In the murine SCI model, grafting of neural stem cells (NSCs) reduced levels of several cytokines produced within the injured areas: TNF-α, IL-1β, IL-6, and IL-12 [58]. In the same model, the NSC-medium reduced the expression of pro-inflammatory cytokines as well as inducible nitric oxide synthase (iNOS) [62]. Mesenchymal stem cells are also well-known regulators of the inflammatory reaction when grafted in various injured tissues as they can release a wide range of bioactive factors (VEGF, BDNF, GDNF, neurotrophin-3/4) or cytokines (GMCSF, MCP1) resulting in the protection/regeneration of injured host tissue or the modulation of immune reactions [64]. Interestingly, the grafted cells are able to adapt their fate and functions to the specific lesion microenvironment, which leads to the secretion of the lesion-induced secretome via transplanted and host cells [65]. MSCs transplanted into SCI downregulated genes are related to cell-cycle regulation/progression, the DNA metabolic/biosynthetic process, and DNA repair, but the upregulated expression of genes is related to cytokine production, phagocytosis/endocytosis, and immune cells/response and thereby adopt immune cell-like characteristics within the injured host tissue [66]. Similarly, grafted undifferentiated mouse iPSCs produced MIP-1α, IL-10, GDNF, and NT-4, resulting in decreased microglial and astrocytic reactions accompanied by significant neuroprotection and functional recovery [67]. The critical role of grafted cells’ secretome has been proven in functional blocking experiments with neutralizing antibodies [68,69]. However, the injury-induced secretome was produced within a relatively narrow time window, namely, as long as the cells remained undifferentiated [67,68]. The understanding of stem cell-induced neuroprotective mechanisms may provide perspectives of new approaches based on cell-free but stem cell-derived products. For example, experiments that employed the delivery of small extracellular vesicles derived from NSCs revealed lowered production of pro-inflammatory cytokines TNF-α, IL-1β, and IL-6 within the host spinal cord tissue [42]. Moreover, the systemic application of exosomes derived from hucMSCs in mice after contusion spinal cord injury upregulated anti-inflammatory cytokines IL-6 and IL-10, while downregulating pro-inflammatory TNF-α, IL-6, IFN-γ, and MIP-1α [57]. Significant improvement of locomotor functions of treated animals was assessed in behavioral or electrophysiological tests [42,57,65,69].

## 3. Keeping a Balance between Neuronal Apoptosis and Autophagy

Apoptosis, as a result of spinal cord injury, affects several groups of cells, including neurons, oligodendrocytes, microglia, and astrocytes [70]. Preventing neuronal apoptosis is one of the expected outcomes of cell transplantation after SCI. Apoptotic cell death is triggered by environmental clues such as oxidative stress and specific molecules secreted post-injury [71] and regulated by the cascade of caspases [72,73]. A mechanism of action based on downregulating the activity of caspase-3 has been reported for neural precursor cells [59], human amniotic mesenchymal stem cells [74], and rat bone-marrow-mesenchymal cells [75] transplanted in rodent SCI models. MSCs have been shown to downregulate caspase-12 [76] and caspase-9 expression, and the largest effect was obtained when co-transplanted with olfactory ensheathing cells [77]. Apoptosis could be inhibited by MSCs directly [75,78], by administration together with selected pharmaceuticals [79], co-transplantation with olfactory ensheathing cells [77], or by their secretome, which was evidenced by the administration of MSC-derived extracellular vesicles into the injured spinal cord [80]. The administration of MSC-derived exosomes reduced the expression of pro-apoptotic factor Bax while elevating the expression of the anti-apoptotic protein Bcl-2, thus promoting recovery in the rat SCI model [81]. Neuronal homeostasis is controlled by numerous tightly regulated mechanisms, and autophagy seems to be one of the most important. Autophagy is a complex process that involves lysosome-dependent removal or recycling of damaged or unnecessary intracellular components, usually leading to the death of dysfunctional cells. On the other hand, under favorable conditions, such intracellular rearrangements may allow cells to adapt to metabolic stress, produce energy, and survive. Autophagy remains in a close relationship with apoptosis. Both mechanisms may be triggered by similar stress factors and they can display a mutual inhibition–activation balance: an induction of autophagy inhibits apoptosis [82] and, in turn, the inhibition of autophagy leads to apoptosis, both in vitro and in vivo [83]. Following spinal cord injury, autophagy is usually disrupted, which results in increased neural apoptosis [84,85]. Therefore, inducing autophagy in affected cells becomes one of the proposed approaches for rescuing neurons and enhancing recovery in murine SCI [86] and traumatic brain injury in rats [87]. It has been shown that the administration of NSC-sEV increased the expression of autophagy factors LC3 and beclin-1, the proteins that promote the formation of autophagosomes and double-membrane vesicles, carrying unwanted cell components to the lysosomes with which they then fuse, releasing their contents for degradation. These direct effects on autophagy were associated with anti-apoptotic action, namely the reduced expression of pro-apoptotic factor Bax, while upregulating anti-apoptotic factor Bcl-2 in host neurons [42].

Another proposed mechanism of the anti-apoptotic action of transplanted cells may be related to mitochondrial transfer from MSCs to the affected motor neurons in the injured spinal cord. Such organelle internalization, shown in vitro and in vivo, improved the bioenergetics of neurons, which could lead to the blockage of apoptotic pathways followed by the facilitation of locomotor recovery [88].

Novel approaches in SCI treatment that are currently being tested in animal models employ the application of non-coding, single-stranded short fragments of RNA (microRNAs) that regulate the expression of many important genes involved in apoptosis. The global miRNA expression profile changes after SCI, showing a downregulation of miR-124, -138, -235-3p, -137, and -30b-3p and an upregulation of miR-1, -15b, -34, and -145. Potential targets of those mRNAs are genes related to apoptosis [89,90]. Engrafting MSC-EVs in a rat SCI model resulted in fewer cell deaths, which is likely a consequence of the miR-21-5p presence in EVs. miR-21-5p was shown to modulate the expression of ligands for the dead receptor Fas, thus downregulating the process of neural apoptosis [91]. On the other hand, overexpression of miRNA-124 in MSCs was shown to drive their differentiation into neurons and promote functional recovery of spinal rats [92]. However, another possible mechanism for this recovery could be the decrease in the number of apoptotic cells; miRNA-124 blocks the mitochondrial pathway of apoptosis in vivo [93] and LPS-induced apoptosis in vitro [94].

## 4. Enhancing Angiogenesis

Vascular disruption is one of the traumatic consequences of spinal cord injury. Due to the alterations of microvessel structures, the blood flow changes and the blood–spinal cord barrier breaks down, which enables enhanced infiltration of inflammatory cells and intraparenchymal hemorrhages. Ultimately, both endothelial and neurons die due to ischemia [95,96] and vascular networks are damaged. Efforts have been made for endogenous revascularization in specific time windows post SCI, but the structure of the vessel network became altered, and the process was not efficient [95,97,98].

Angiogenesis, measured by the increasing density of blood vessels, is stimulated via the bioavailability of angiogenic factors such as vascular endothelial growth factor (VEGF). Therapeutic approaches for SCI therefore mainly focus on enhancing the VEGF local concentration to support angiogenesis and restore vascular perfusion [96]. However, during post-SCI wound healing, blood vessels display altered barrier properties, such as different vessel permeability and altered distribution of glucose transporters. The newly formed vessels with higher permeability can be leaky for inflammatory cells and thus increase neuroinflammation. The direct injection of exogenous VEGF in the rat SCI model has been reported to produce significant microvascular permeability, higher infiltration of leukocytes into the spinal cord parenchyma as well as exacerbation of secondary damage [99]. Moreover, when administered alone, the angiogenic factors might initiate angiogenesis but are not able to support blood vessel maturation. Thus, promoting stabilization of the angiogenic response and blood vessel maturation seems to be a better approach to improve recovery. A combined administration of VEGF with the platelet-derived growth factor (PDGF), known for promoting blood vessel maturation and stability, significantly reduced lesion size and gliosis after hemisection of the spinal cords in rats [100].

Such beneficial effects can be reached by other strategies, among which there is the transplantation of angiogenic growth factor-producing cells. Promising results were obtained when engrafting olfactory ensheathing cells alone or in co-transplantation with adipose-derived stromal cells, which secrete proteins involved in VEGF pathways [101]. However, due to the relatively low survival and integration rate, the satisfactory functional outcome of OECs transplantation has not been reported to date [102]. The generation of new vessels in the injury site can be also reached by transplantation of NSCs, either naïve or genetically manipulated to overexpress VEGF, eventually leading to improvement in locomotor functions in the rat model of SCI [103,104]. Furthermore, iPSCs-derived neurospheres [105], MSCs-derived exosomes, or an MSC-conditioned medium were shown to secrete proangiogenic factors such as VEGF, FGF2, and angiogenin. This effect was observed both in vitro and in vivo, where the newly formed vessels were found in rat injured spinal cords after treatment with MSC exosomes [106,107].

Angiogenesis can be modulated with the aid of synthetic and biomaterials, which are applied in the injury site [108]. There are many materials and strategies developed, among which there are procedures that directly deliver angiogenic factors to the lesion site. Proangiogenic VEGF, angiopoietin-1, and bFGF encapsulated in biocompatible microspheres enhanced vascularization and motor functions after SCI in rats [109]. Similar results were obtained with collagen scaffolds delivering VEGF directly into the lesion site [110]. Furthermore, intraspinally applied scaffolds can also mimic the extracellular matrix, and when grafted together with specific cell types, promote their adhesion, survival, and proliferation. Experimentally, transplantation of human MSCs incorporated either in synthetic or bio-scaffolds into rat injured spinal cord resulted in enhanced neovascularization via VEGF and HIF-1α expression [111]. Another growth factor that contributes to angiogenesis is the hepatocyte growth factor (HGF). HGF has been shown to improve microcirculation and angiogenesis when introduced into injured spinal cord tissue by viral infection [112] or intrathecal injection alone or together with a modified gelatin scaffold [113,114]. Functional recovery through HGF induction was also observed in aged mice after neural stem cell transplantation [115]. Nevertheless, there is still not much research focusing on cell therapies and their impact on the HGF pathway, therefore their effectiveness needs to be assessed.

## 5. Increasing Axonal Sprouting and Regeneration

During spinal cord injury, high-power mechanical forces often cause nerve tissue damage via crushing or tearing. It usually results in the disruption of axons running along the spinal cord. Mechanical damage often leads to secondary injuries via so-called Wallerian degeneration, which is defined as a progressive anterograde disintegration of axons and accompanying demyelination after an injury to the proximal axon or cell body [62]. On the other hand, inflammatory reaction resolution and the scar formation produce an unfavorable environment for the growth and regeneration of axons. Therefore, it is likely that corticospinal axon sprouting and regeneration after SCI might be enhanced by either providing a permissive environment for their growth or, on the other hand, neutralizing growth inhibitory molecules or preventing axons from recognizing them, which would allow axons to elongate into otherwise hostile surroundings. The ability of neural progenitor cells to secrete a variety of neurotrophic factors indicates that they could promote axonal growth either by providing a guiding substrate for axon regeneration or preventing/removing the existing scar.

To remove the already formed scar, chondroitinase ABC, the enzyme digesting chondroitin sulfate and the main component of the glial scar, may be employed [116]. Degradation of the inhibitory extracellular matrix molecules promotes robust sprouting of spinal projections in injured areas of the spinal cord, which results in structural plasticity and functional recovery [117]. Moreover, chondroitinase treatment has been shown to significantly increase corticospinal axon growth and the number of synapses formed by corticospinal terminals in grey matter caudal to the lesion in rhesus monkeys. Hand function was improved significantly in treated animals compared to vehicle-injected controls, with no detrimental effects detected. This approach appears to merit clinical translation in spinal cord injury [118].

The enzymatic approach produces particularly good results in combination with other procedures, such as simultaneous peripheral nerve transplantation [119], hyperbaric oxygen therapy [107], laser irradiation [120], or transplantation of different type of exogenous cells [121,122] or treadmill training [123]. An approach of intrathecal pretreatment with chondroitinase ABC before the transplantation of induced pluripotent stem cell-derived neural stem cells (iPS-NSCs) in mice subjected to spinal cord injury contributed to glial scar reduction [122]. What is more, this strategy significantly increased the survival of transplanted cells and their differentiation into three neuroglial lineages. The patch-clamp analysis showed that neurons differentiated from transplanted iPS-NSCs integrated with spinal cord circuits by forming functional synapses with host neurons. Consequently, animals showed functional recovery manifested by higher forelimb grip strength and better locomotion ability measured by automated Catwalk gait analysis [122].

However, it was shown that chondroitinase ABC loses its enzymatic activity very quickly at body temperature, which significantly limits its application in human SCI therapy [124]. Therefore, genetically modified cells have been developed to constantly release this enzyme when transplanted intraspinally. For example, canine olfactory ensheathing cells continuously producing chondroitinase ABC transplanted in rat crushed dorsal columns allowed an increase in axonal sprouting within the corticospinal tract rostral to the lesion and the number of corticospinal axons caudal to the lesion site [125]. Similar effects were also obtained in other studies showing that the transplantation of genetically modified Schwann cells, simultaneously producing chondroitinase ABC and neurotrophin D15A, led to axon regeneration and improvement of movement ability (higher BBB score) of rats after thoracic contusion injury [126].

The presence of astrocytes within the affected tissue might also be, however, beneficial to the regenerating axons. Regeneration of the injured axons is very often observed along “bridges” created by endogenous astrocytes [127], thus glial progenitor transplantation is expected to be an effective strategy for SCI treatment by creating the permissive environment. Indeed, it has been reported that intraspinal transplantation of glial progenitor cells in rats after SCI resulted in improved sensory and motor (reticulospinal and raphespinal) tract regeneration that was manifested by enhanced axonal growth [128]. Another study showed that astroglial progenitors, transplanted after cord hemisection, survived for 5 weeks in the host spinal cord and differentiated into astrocytes, leading to better regeneration of the descending rostral–ventral respiratory group of axons. The improvement of the respiratory functions with a higher amplitude of diaphragm electromyographic activity was also observed [129]. One of the mechanisms responsible for such beneficial effects could come from the delivery of certain specific, astrocyte-derived proteins. For example, it has been shown that periostin is highly expressed by bone morphogenetic protein-induced astrocytes derived from embryonic glial-restricted precursors (GDAs^BMP^), and the decrease in its expression by shRNA abolished GDAs^BMP^-induced axon outgrowth in vitro. Furthermore, transplantation of POSTN-deficient GDAs^BMP^ into the injured rat spinal cord resulted in lower axonal regeneration. On the other hand, the addition of recombinant periostin to neurons in vitro abolished the adverse effects of glial scar molecules and increased neurite extension via the influence on FAK and Akt pathways [130].

Axonal regeneration could be also promoted indirectly. It has been shown that intraspinal transplantation of oligodendrogenic neural progenitor cells in nude rats after spinal cord injury led to the functional recovery of injured animals measured by BBB scores and Catwalk analysis of locomotor functions. In this case, transplanted cells mainly differentiated into oligodendrocytes, which promote tissue sparing and axon remyelination [131]. NPC can be genetically modified to secrete factors that contribute to lesion healing. One of them is erythropoietin (EPO), which has well-documented neurotrophic and neuroprotective properties [132,133] and has been extensively examined for perspective SCI treatment [134,135]. Experiments in mice subjected to SCI revealed that EPO-releasing NPCs (Er-NPCs) accumulated, survived, and differentiated within the lesion area, which led to enhanced axon regeneration and 5-HT innervation, resulting in the improvement of locomotion abilities of tested animals [136]. Another protein with a reported beneficial effect in SCI is fibroblast growth factor 2 (FGF2). Intraspinal transplantation of dental pulp stem cells primed with FGF2 in rats immediately after thoracic total spinal cord transaction led to significant improvement of the locomotor ability of tested animals (measured by the increase in BBB score) in comparison to not-grafted or only DPSC-grafted animals. Of note, DPSCs appear to be particularly useful for transplantation since they can be very easily derived from various dental tissues during routine dental procedures and can differentiate into many cells such as liver cells and β cells of islet of the pancreas, cardiomyocytes, chondrocytes, osteoblasts, and neurons, which provides great potential to use them in regenerative medicine [137], including SCI treatment [138]. Additional analysis showed that FGF2 release protected transplanted cells from death induced by reactive oxygen species in the injured tissue. Surviving DPSCs promoted axonal regeneration and locomotor function recovery [139].

Molecules instructive for signaling pathways involved in axon growth, such as microRNAs, could also be employed to modulate axon survival after injury. Intravenous application of exosomes delivered from stem cells expressing miR-133b in rats after compression spinal cord injury led to an increase in axon regeneration and improvement of their locomotor ability [140]. The observed beneficial effect was mainly associated with decreased expression of the Rho A protein, which participates in axon growth cone degradation and influences CREB and STAT3 pathways [140].

## 6. Supporting Remyelination

Myelin is a lipid-rich structure produced and wrapped around axons to protect them and enable fast impulse propagation [141]. SCI affects myelin integrity via the necrosis and apoptosis of oligodendrocytes, the myelin-producing cells of the CNS. Oligodendrocyte death is caused by several factors such as cytotoxic cytokines or reactive forms of oxygen/nitrogen formed after injury. Demyelination contributes to further damage, interfering with the transduction of impulses by denuded axons, which become ultimately necrotic [142,143]. However, myelin sheaths may be restored spontaneously to demyelinated axons. Following an injury to the white matter, oligodendrocyte precursor cells (OPCs) are activated and recruited to the lesion, change the morphology, and upregulate the expression of many genes, which are further essential to their successful differentiation into oligodendrocytes, forming new myelin [144,145].

**Figure 1:**
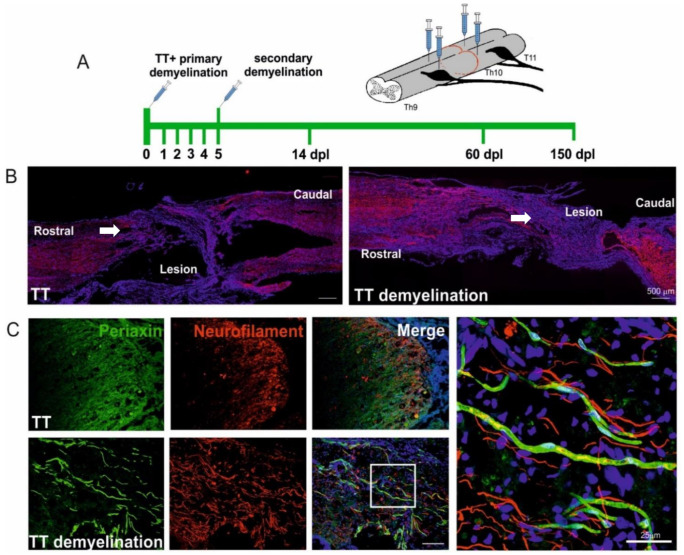
Experimentally induced white matter remyelination supports spontaneous axon growth in the transected spinal cord. (**A**). Schematic description of experimental design. Focal demyelination was induced by stereotaxic injection of glial toxin bilaterally into the dorsal and ventral funiculi as well as both rostrally and caudally to the transection site using the method described by Woodruff and Franklin [149]. Note the defined phases of experiments: Double focal demyelination (first at the day of total transection TT and then 5 dpl), histological examination at the early (14 dpl) and complete remyelination (60–90 dpl) and functional examination (150 dpl). Five adult female WAG rats were used in experimental (transection combined with demyelination) and control (only transection) groups. (**B**). In the rat spinal cords after total transection combined with demyelination we found a significantly larger number of more longitudinally oriented axons distributed at the adjacent area of the lesion large amount as well as axons crossing the spared tissue at the epicenter of the lesion, compared to control animals without demyelination induced (neurofilament-immunopositive fibers in red, cell nuclei in blue). Scale bars 500 μm. (**C**). Representative images showing Schwann cell-derived remyelination of axons growing within the injured area in close proximity to the transection site (marked with arrows in B), Schwann cell-specific myelin protein, Periaxin, in green, axons neurofilaments in red, cell nuclei in blue. Scale bars 25 μm. The animal procedures were performed according to the guidelines from Directive 2010/63/EU of the European Parliament on the protection of animals used for scientific purposes.

Spontaneous myelin regenerative events often occur after SCI, indicating that the spinal cord attempts to repair itself. In rodent models of experimental SCI, oligodendrocyte progenitors repopulate the affected area and remyelinate intact demyelinated axons [146]. After spinal cord injury, when glia limitans are destroyed, Schwann cells of the PNS-origin also easily migrate from spinal roots to the lesion, interact with demyelinated CNS axons, and myelinate them. What is more, OPCs are also able to differentiate into Schwann cell-like cells producing myelin of the PNS type, myelinating and regeneration-promoting within the CNS [145,147,148]. Remyelination, regardless of whether it is driven by oligodendrocytes or Schwann cells, results in increased expression of regeneration-associated genes in injured neurons and ultimately saves them from death. Our recent findings suggest that experimentally induced white matter remyelination creates a unique environment within the transected spinal cord that is permissive for spontaneous axon growth and could be promising in achieving substantial functional improvement (Figure 1).

Unfortunately, spontaneous remyelination in the CNS often fails or is incomplete, and non-myelinated axons can be observed months after SCI in rat models [131,150,151]. Whether endogenous remyelination is efficient enough for axon regeneration and whether enhancing myelination from exogenous sources would be a good strategy for SCI has become a major topic in the ongoing debate [143,150,152]. Nevertheless, it appears necessary to understand the mechanisms of this unique regeneration process and the significance of its contribution to SCI repair in order to develop novel therapeutic approaches targeting remyelination.

Several potentially therapeutic strategies for enhancing remyelination in SCI treatment have been already experimentally examined. One of the promising approaches is a direct method of replacing oligodendrocytes lost during injury by grafting OPCs of different origins. OPCs derived from human embryonic stem cells intraspinally grafted in rats 7 days after spinal cord contusion survived in the spinal cord tissue, redistributed over short distances, and differentiated into oligodendrocytes, successfully enhancing remyelination. Functional improvement manifested as longer forelimb stride length, increased forelimb step range, and a higher number of passed-perpendicular steps as measured by kinematic analysis. What is more, rats exhibited motor behavioral recovery up to 4 months after transplantation with no significant toxicities observed [153,154]. It has been shown that iPSC-derived OPCs engrafted into the injured spinal cord were also able to differentiate into mature oligodendrocytes in a rather promising quantity of more than 70% of engrafted cells. They myelinated axons and promoted recovery [155]. An alternative way to elevate the number of oligodendrocytes after injury might be engrafting neural progenitor/stem cells that can be derived from either iPSC or other adult tissue sources [156,157,158]. Since only about half of NPCs differentiate into oligodendrocytes in the injury site [159], cells can be reprogrammed in vitro for a more a oligodendrogenic fate prior to transplantation [131,160] to generate more oligodendrocytes, which increases myelination. One of such approaches is creating NSCs lines with the overexpression of transcription factor Olig2, a determinant for OPC formation from NSCs [161].

Positive effects were observed after the experimental grafting of SCs into the injured spinal cord as well. Given the fact that autologous cells are difficult to obtain, SCs can be derived from other sources, including bone marrow and skin-precursor cells [162,163]. Better outcomes and functional recovery can be obtained when engrafting genetically modified Schwann cells, often in cooperation with other cell types. Schwann cells, modified to overexpress either GDNF or neurotrophin together with chondroitinase, which degrades glial scar, enhanced myelination and axonal survival [126,164]. Promising results were achieved when SCs, overexpressing NGF and/or BDNF, were co-transplanted with fetal spinal cord cells or NSCs [165,166]: SC-derived factors enhanced oligodendrocyte maturation and myelination. Recently, skin-derived precursors differentiated into Schwann cells (SKP-SCs) were transplanted directly into the contused rat spinal cord acutely [167] or several weeks post-injury [162]. SKP-SCs survived transplantation well, integrated with host tissue filling much of the lesion sites, and greatly enhanced the presence of endogenous SCs, which myelinated thousands of host axons in the area of injury. This approach is perhaps the most clinically relevant one to date and is promising for the treatment of chronic SCI since it resulted in an improved locomotor outcome that was very rarely observed with cell transplantation beyond the sub-acute stage of injury.

## 7. Rebuilding and/or Activation of Spinal Cord Network

Neural necrosis and apoptosis destroy the innervation derived from the higher supraspinal structures that activate and modulate the activity of spinal cord networks located below the injury site. Thus, the reorganization of local neuronal circuits through the repopulation of damaged tissue with the newly generated neurons is essential for the restoration of lost function.

A range of cell types has been experimentally examined as a potential source of neurons to replace the lost populations. Intraspinal transplantation of motoneuron-like cells, pre-differentiated from human adipose-derived stem cells in vitro, has been reported to significantly improve locomotor abilities in mice after crushing SCI [168]. Transplanted cells survived in the host spinal cord and functionally integrated with the network, which was confirmed by electrophysiological experiments; the whole-cell patch-clamp in vitro analysis has proved the proper motoneuron characteristics of differentiated motoneuron-like cells. The motor-evoked potentials were elicited in the frontal cerebral cortex and recorded in the skeletal muscle of the hindlimb. It has been also shown that iNSCs produced in vitro from embryonic murine fibroblasts transplanted into the injury site of SCI rats resulted in functional recovery. The effect was manifested by better locomotor ability (a higher BBB rate and lower horizontal ladder test score rate) and bladder function (return of normal bladder volume level and normal voiding pattern). Immunohistochemical analysis showed that transplanted iNSCs survived in the host spinal cord and differentiated into neurons, astrocytes, and oligodendrocytes. Most iNSCs-delivered neurons were either GABAergic, glutamatergic, or cholinergic. Neurons differentiated from transplanted cells created functional synapses with spinal cord neurons and were integrated with the host spinal cord network, leading to supraspinal connection regeneration. Moreover, transplantation of iNSCs may be even more beneficial since the cells were reported to be involved in increased angiogenesis and inhibition of the inflammatory response [169].

An optimistic notion that certain lost spinal cord connections could be successfully replaced has been recently strongly supported by results of pre-clinical primate experiments showing that human spinal cord-derived multipotent NPCs transplanted at 4 weeks after cervical hemisection in rhesus monkeys gradually maturated and improved forelimb function [170]. Human cells survived in the host spinal cords and extended large numbers of axons over long distances, finally forming reciprocal synaptic connections with host circuits caudally to the lesion. Kumamaru et al. [171] demonstrated that spinal NPCs grafted to injured spinal cord give rise to a variety of neuronal progeny representing most neuronal subtypes of the intact spinal cord in both rat and nonhuman primates. Importantly, successful engraftment resulted in increased corticospinal tract axon growth across human cell grafts, indicating the ability of host axons, also essential for voluntary movement, to be regenerated. Moreover, improved forelimb function, as well as long-lasting neuroplasticity effects, were observed. These findings indicate that homologous neural stem cells might enable regeneration of the corticospinal projection within and beyond spinal cord lesions [172]. Remarkably, regenerating host corticospinal motor axons were shown to specifically innervate appropriate pre-motor interneurons and avoid inappropriate sensory targets within the grafted area, without a need for additional exogenous guidance. These findings provide strong evidence that injured adult axons retain the ability to recognize appropriate targets and avoid inappropriate ones within “replaced” areas, suggesting that the restoration of complex circuitry after SCI may be achievable. The latter is particularly important for the restoration of the voluntary motor-control system in humans and might be potentially beneficial in clinical translation. These reports also highlight the importance of utilizing nonhuman primate models in order to reduce the risk of clinical trial failure due to graft loss [171].

SCI very often leads to the disruption of supraspinal GABAergic inhibitory projections, which innervate interneurons in I-III lamina of the spinal cord dorsal horn [173], leading to increasing neuronal excitability and the development of neuropathic pain [174]. Therefore, transplantation of GABAergic neurons to restore the inhibitory input to the dorsal horn disrupted after SCI is one of the postulated methods for the treatment of neuropathic pain. Intraspinal transplantation of GABAergic neurons differentiated from murine ESCs in a rat SCI pain model led to a reduction of neuropathic pain observed in injured animals, which was manifested by the abolition of hemisection-induced tactile hypersensitivity and neuronal hyperexcitability [175]. Histological analysis showed that transplanted cells survived in the host spinal cord and localized mainly in the lower-medial part of the dorsal funiculi, in the spinal white matter. Application of GABA A and GABA B receptor antagonists, in contrast to the serotonergic receptor antagonist, abolished the beneficial effect of cell transplantation in injured animals, showing a specific restoration of spinal GABAergic innervation by transplanted cells. Similar results were also obtained in an experiment in which embryonic stem cell-derived GABAergic neural precursor cells were transplanted into rats two weeks after contusive SCI [176].

**Figure 2:**
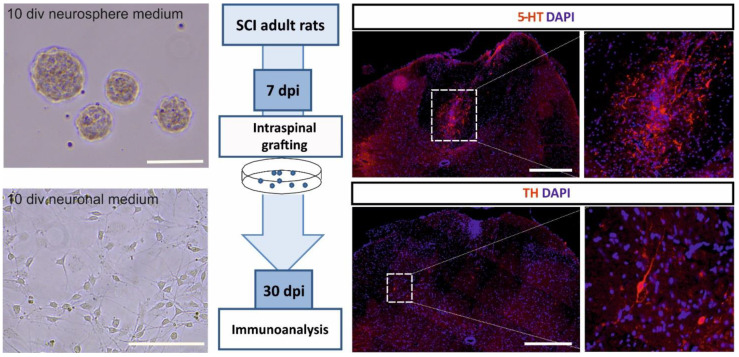
Schematic presentation of the results of preliminary experiments showing serotonergic precursors surviving and terminally differentiating after transplantation into the spinal cord injury-mediated environment. The left panel shows precursor morphology in different cell culture conditions: Neurosphere medium supports cell proliferation and neurosphere formation, neuronal medium induces their neuronal differentiation. Scale bars 100 μm. The middle panel describes the experimental design: Expanded serotonergic precursors derived from rat embryonic brain stem (on embryonic day 11) were transplanted into the rat injured spinal cord 7 days post-injury and their morphology was examined after the next 30 days. The protocol for cell expansion was adopted from [196]. The right panel shows representative images of differentiated cells within host spinal cords. Transplant-derived serotonergic neurons were found in each examined injured spinal cord (three rats). Note the presence of serotonin (5-HT)-positive neurons (upper images) and thyrosine kinase (TH)-positive neurons (lower images). Control animals were injected with cell culture medium, no stained cells were detected (data not shown). Scale bars are 500 μm. Functional experiments that examined the recovery of locomotor functions via restored innervation of the spinal cord are in progress.

Many reports contribute to building the growing body of evidence that both noradrenergic [19,177,178] and serotonergic [18,20,179,180,181,182,183,184,185,186,187] innervation play an important role in the control of locomotion. Noradrenergic and serotonergic neurons that send projections to the spinal cord are located in *locus coeruleus* and *locus subcoeruleus* (noradrenergic neurons) [188,189] and three nuclei of the brain stem: *n. raphe pallidus*, *n. raphe obscurus*, *n. raphe magnus* [190], and the parapyramidal region (serotonergic neurons). These innervations are often disrupted after SCI, so one of the postulated methods for SCI treatment is restoring monoaminergic innervation of networks responsible for locomotor pattern generation that survive below the injury site. Historically, it has been shown that embryonic brain tissue transplants containing noradrenergic neurons from *Locus ceruleus* [191] or serotonergic cells from the brain stem [191,192] transplanted to the spinal cord survived in the host tissue and innervated the proper areas of the host spinal cord. Intraspinal transplantation of 14-day-old rat embryo brain stem tissue containing serotonergic areas into adult rats after thoracic spinal cord total transaction resulted in improved treadmill locomotor ability of injured animals, which is shown by kinematic and electromyographic analysis [187,193,194]. The beneficial effects were abolished by application of antagonists for both 5-HT_2_ [186,195] and 5-HT_7_ [186] serotonin receptors, suggesting that those receptors play a pivotal role in the modulation of spinal cord networks and restoration of the locomotor ability after SCI. We also demonstrated the role of noradrenergic neurons present in the intraspinally grafted embryonic brainstem tissue for the recovery of locomotor hindlimb function of paraplegic rats [181]. Recently, we expanded serotonergic precursors derived from embryonic brain stem in a neurosphere assay in vitro and transplanted them into the rat spinal cord one segment below total transection. The results of our current studies provide further evidence that serotonergic precursors survive the transplantation and terminally differentiate within the host environment (Figure 2). However, it remains to be examined whether the transplantation of pure serotonergic neurons would be just as, or more, effective as the embryonic brain stem tissue transplantation.

## 8. Concluding Remarks

In recent years, extensive experimental work has been devoted to understanding the mechanisms underlying neuroprotective or regenerative outcomes of cell transplantation in neurological conditions such as spinal cord injury. Despite the advances in uncovering the regenerative mechanisms triggered by exogenous cells, certain long-lasting issues still need to be resolved. Reviewing the papers listed here, we focused our attention on the cell-based approaches that ensure significant functional regeneration of the injured spinal cord and provide some facilitation in motor function recovery. Although several methods (like BBB or BMS) were created to achieve a level of standardization of locomotor performance evaluation, they might be not sufficient to assess certain important changes in the locomotor pattern, such as intra- and interlimb coordination and their relation to locomotor speed, particularly in the chronic stage of injury in long-lasting experiments. The majority of attempts reported to date show very slight improvement of locomotor function, particularly after total spinal cord transection/complete injury or severe spinal cord contusion. In many cases, the locomotor improvement is limited to better movement in individual joints without any better limb coordination or plantar stepping and body weight support. Since the majority of SCI patients suffer from severe complete or incomplete loss of function as a consequence of cervical spinal segments injury, it seems that more efforts are still required to adapt the experimental situation to clinical needs.

As shown above, an increasing number of data from experimental and pre-clinical studies has been generated to support the notion that exogenous cell transplantation promotes repair in the SCI and could be the most promising translational pathway. Nevertheless, the majority of pre-clinical cell-based studies were performed with rodent models and some of them have already reached early-phase human clinical trials. However, the current revision of the registered trials revealed that the overwhelming majority of them remain at an early stage [197]. Ninety-six percent of them are either phase I, phase II, or nested phase I/II designs, with the significant majority being single-site studies. Of forty-nine registered trials, the majority used autologous cells, primarily using stem cells, mostly MSCs, although a smaller number used Schwann cells or olfactory ensheathing cells, with mixed results. No NSC trials have yet shown sufficient efficacy, but four out of the five published examples have reported some functional improvement [197].

Although different aspects of SCI pathology and many of their overcoming mechanisms via cell-based treatment have been experimentally examined and successfully assessed in animal models, despite the initial enthusiasm it sparked, their implementation to routine clinical practice is notably limited to date. Since the chronic treatment of spinal cord injury is particularly challenging, it became evident that the capacity of these approaches to facilitate regeneration and achieve meaningful functional recovery is rather modest when considered individually. Therefore, it is now widely postulated that combined therapeutic strategies targeting different aspects of SCI pathology and acting complementary could likely offer more satisfactory functional outcomes and should be clinically tested.

## Data Availability

Not applicable.

## Abbreviations

5-HT	5-hydroxytryptamine, serotonin
5-HT_2_	5-HT_2_ receptors for serotonin
5-HT_7_	5-HT_7_ receptors for serotonin
AMPA	α-amino-3-hydroxy-5-methyl-4-isoxazole propionic acid
Bax	pro-apoptotic Bcl-2 protein factor
BBB	Basso-Beattie-Bresnahan locomotor rating score
Bcl-2	anti-apoptotic protein inhibiting Beclin 1-dependent autophagy
BDNF	brain-derived neurotrophic factor
BM-MSCs	bone marrow mesenchymal stem cells
BMS	Basso Mouse Scale for locomotor rating
CNS	central nervous system
CREB	cAMP response element-binding protein div days in vitro
dpl	days post lesion
DPSCs	dental pulp stem cells
EPO	erythropoietin ESC embryonic stem
FAK	focal adhesion kinase
FasL	ligand for Fas receptor
FGF2	fibroblast growth factor 2
GABA	gamma-aminobutyric acid
GDAs^BMP^	bone morphogenetic protein-induced astrocytes derived from embryonic glial-restricted precursors
GDNF	glial cell-derived neurotrophic factor
GMCSF	granulocyte-macrophage colony-stimulating factor
HGF	hepatocyte growth factor
HIF-1α	hypoxia-inducible factor 1-alpha
IFN-γ	interferon γ
IL-10	interleukin 10
IL-12	interleukin 12
IL-1β	interleukin 1β
IL-6	interleukin 6
iNOS	inducible nitric oxide synthase
iNSCs	inducible neural stem cells
iPSCs	inducible pluripotenet stem cells
iPS-NSCs	induced pluripotent stem cell-derived neural stem cells
iPSs	induced pluripotent stem cells
KA	kainic acid
LC3	autophagy factor
M1	pro-inflammatory type of macrophages
M2	anti-inflammatory type of macrophages
MAG	myelin-associated glycoprotein
MCP1	monocyte chemoattractant protein 1
MIP-1α	macrophage inflammatory protein 1-alpha
MSCs	mesenchymal stem cells
NF-κB	nuclear factor kappa B
NGF	nerve growth factor
NMDA	N-methyl-D-aspartate glutamate
Nogo-A	neurite outgrowth inhibitor A
NSCs	neural stem cells
NPCs	neural progenitor cells
NT-4	neurotrophin-4
Olig2	oligodendrocyte transcription factor
OECs	olfactory ensheathing cells
OPCs	oligodendrocyte precursor cells
PNS	peripheral nervous system
RhoA	ras homolog family member A
ROCK	Rho-associated protein kinase
SCI	spinal cord injury
SCs	Schwann cells
sEV	small extracellular vesicles
SKP-SCs	skin-derived precursors differentiated into Schwann cells
STAT3	signal transducer and activator of transcription 3
TH	tyrosine hydroxylase
TNF-α	tumor necrosis factor-α
TT	total spinal cord transection
VEGF	vascular endothelial growth factor
Wnt/β	catenin
